# A “New Way to Take the Temperature”

**Published:** 1886-10

**Authors:** E. L. Sessions

**Affiliations:** Whitney, Texas


### A "NEW WAY TO TAKE THE TEMPERATURE" (?).

EDITOR DANIEL'S TEXAS MEDICAL JOURNAL:

GOING the rounds of the journals lately, is an article entitled a "New Way to Take the Temperature Quickly," thus: "Warm the instrument, and then observe the degree of fall instead of rise." The writer of the article asserts that the thermometer may be placed in the axilla, and not more than two minutes will give you an accurate record of the temperature. I would like to know how an index to a self-registering thermometer can be made to act so absurdly as to rise or fall at the will of the physician? The alleged plan might work with one of the old style thermometers; that is, one in which the mercury commences falling as soon as it is taken from a warm place to a cooler atmosphere; or, in other words, a thermometer that has no self-registering index, or one that had been ruined by the mercury in the bulb and the index getting together. I write this merely to get information, as, perhaps, I am stupid and unable to see it, as the paragrapher intended.

Yours truly, E. L. SESSIONS.

Whitney, Texas, September 20.

